# The Impact of Air Pollution Information on Individuals’ Exercise Behavior: Empirical Study Using Wearable and Mobile Devices Data

**DOI:** 10.2196/55207

**Published:** 2024-09-10

**Authors:** Yang Yang, Khim-Yong Goh, Hock Hai Teo, Sharon Swee-Lin Tan

**Affiliations:** 1 School of Business and Management Royal Holloway, University of London Egham United Kingdom; 2 School of Computing National University of Singapore Singapore Singapore

**Keywords:** air pollution, information sources, exercise activity, wearable and mobile devices, econometric analysis

## Abstract

**Background:**

Physical exercise and exposure to air pollution have counteracting effects on individuals’ health outcomes. Knowledge on individuals’ real-time exercise behavior response to different pollution information sources remains inadequate.

**Objective:**

This study aims to examine the extent to which individuals avoid polluted air during exercise activities in response to different air pollution information sources.

**Methods:**

We used data on individuals’ exercise behaviors captured by wearable and mobile devices in 83 Chinese cities over a 2-year time span. In our data set, 35.99% (5896/16,379) of individuals were female and 64% (10,483/16,379) were male, and their ages predominantly ranged from 18 to 50 years. We further augmented the exercise behavior data with air pollution information that included city-hourly level measures of the Air Quality Index and particulate matter 2.5 concentration (in µg/m^3^), and weather data that include city-hourly level measures of air temperature (ºC), dew point (ºC), wind speed (m/s), and wind direction (degrees). We used a linear panel fixed effect model to estimate individuals’ exercise-aversion behaviors (ie, running exercise distance at individual-hour, city-hour, or city-day levels) and conducted robustness checks using the endogenous treatment effect model and regression discontinuity method. We examined if alternative air pollution information sources could moderate (ie, substitute or complement) the role of mainstream air pollution indicators.

**Results:**

Our results show that individuals exhibit a reduction of running exercise behaviors by about 0.50 km (or 7.5%; *P*<.001) during instances of moderate to severe air pollution, and there is no evidence of reduced distances in instances of light air pollution. Furthermore, individuals’ exercise-aversion behaviors in response to mainstream air pollution information are heightened by different alternative information sources, such as social connections and social media user-generated content about air pollution.

**Conclusions:**

Our results highlight the complementary role of different alternative information sources of air pollution in inducing individuals’ aversion behaviors and the importance of using different information channels to increase public awareness beyond official air pollution alerts.

## Introduction

### Background

Air pollution remains an extremely serious environmental and health hazard [1]. Although exercising can improve one’s health, doing so in polluted air can offset the health benefits and be detrimental to one’s cardiovascular and respiratory health [2]. Providing air quality alert information has become an important initiative adopted by government authorities to protect the public from the risk of air pollution [3], with the anticipation that individuals will adjust their behaviors considering this information. Despite the availability of public health information, there is limited evidence to suggest whether individuals truly act upon this information and the extent to which they may adjust their outdoor exercise behaviors. Furthermore, with government agencies providing more detailed information, concerns have arisen over the danger of air pollution alerts losing their prominence, which can diminish the efficacy of information provision as a policy tool [4]. Thus, it is of immense interest to uncover individuals’ actual reaction and to what extent individuals adjust their exercise behaviors in response to air pollution information.

Air quality alert information helps individuals to maximize the utility of their outdoor activities while accounting for the health risk from air pollution. Prior studies on the relationship between air pollution information and avoidance behavior primarily focus on the impact of mainstream or official air quality information sources [3,4]. Mainstream or official information on air pollution typically include particulate matter (PM) 2.5 concentration and Air Quality Index (AQI) which indicate the severity and potential health hazards of air pollution. In most countries, public agencies monitor and disseminate such information about a city’s pollution parameters through AQI announcements on television, radio, newspaper, and website channels.

Beyond the mainstream sources, there are alternative information sources through which individuals can access air pollution information. Particularly, with the rise of social media, people can access alternative information on air pollution shared by the public on social media platforms and mobile chat apps [5]. Research has shown that social media messages are strongly correlated with AQI and are indicative of true particle pollution levels [6]. Despite the increasing impact of user-generated content on social media [7] and the availability of other information sources (eg, web–based social connections and mobile apps), there is a lack of knowledge in the literature on whether these alternative information sources may moderate (ie, substitute or complement) the effect of mainstream pollution information sources on individuals’ exercise aversion behaviors.

Furthermore, current understanding of the impact of air pollution on individuals’ exercise behavior is limited, and existing research evidence is mainly built on self-reported survey data [8]. These data can suffer from recall errors and social desirability bias [9], and is limited to provide detailed spatial and temporal attributes of exercise behaviors. However, air pollution levels can vary across different specific locations and fluctuate across time. Thus, it becomes critical to provide scientific evidence using real-time, disaggregate spatial and temporal data to understand the extent to which individuals avert exposure to polluted air in their physical activities in real time (ie, on an hourly basis), in response to different sources of air pollution information. According to recent reports on personal wellness and fitness, there is a rise in consumers’ adoption of digital health services and technology-driven fitness programs. More consumers are also using wearable activity trackers, such as smart wristbands and fitness apps, which offer instant feedback on various exercise metrics [10]. Data from these wearable fitness devices and applications enable researchers to address the limitations previously mentioned.

### Objective

We aimed to evaluate the extent to which individuals avoid polluted air during exercise activities, in response to different air pollution information sources. We also examined whether alternative air pollution information sources moderate the effect of mainstream information sources on individuals’ exercise behaviors.

## Methods

### Data

In China, air pollution continues to persist at a concerning level and affects the economy and people’s overall quality of life [11]. Research has shown that air pollution in northern regions of China led to a 5.5 year reduction in life expectancies of residents, compared with those in southern regions [12]. The alarming rate of air pollution makes China a valid and representative context to investigate the research questions.

We conducted empirical analyses using a novel data set of over 16,000 individuals’ running exercise behaviors in 83 Chinese cities over a 2-year time span from January 2013 to November 2014, as captured by wearable devices and mobile fitness apps. This sample period is particularly appropriate because there was a confluence of multiple episodes of severe air pollution in China and the mass adoption of wearable and mobile devices from 2013 to 2014. The data on individuals’ exercise activities were obtained from a China-based, web and mobile fitness platform that offers products (eg, smart wristband and mobile fitness apps) for tracking exercise activities which incorporate social network features. Users connect to the platform through a wearable device, web browser or mobile app, which enables them to track exercise activities, share their progress with peers, and leverage social networking features to foster support and encouragement among users. All data generated during physical exercise activities are synchronized to the platform’s data servers and is viewable by users through the mobile app and website. This individual-level minute-by-minute exercise behavior data was further supplemented with hourly weather controls data and various air pollution information sources such as AQI and PM2.5 measures, social connections, Weibo tweets (a Chinese Twitter-like platform), and mobile apps.

Our exercise behavior data set includes information on individuals’ user anonymous ID, sex, total cumulative distance (km) of exercise activity, reward points balance, exercise skill grade level, home and exercise city location, exercise distance (km), exercise speed (km/h), exercise type (eg, running or walking), exercise tracking date and time (for both start and end of exercise session), tracking device type (eg, smart wristband or mobile fitness app), and calories expended. We removed, from our raw data set, exercise records or observations that had various issues such as duplicated records, missing exercise and home city locations, and outliers in terms of exercise distances (ie, top and bottom 0.5 percentiles, corresponding to likely errors in distances recorded that were >100 km or <0.01 km, for example). We focused only on running and excluded walking, as our data set cannot differentiate whether a physical activity was conducted indoor or outdoor, and walking can occur indoors from point to point. In contrast, running is a recreational physical activity that is mostly conducted outdoors in China [13], and thus is susceptible to the effects of air pollution. Our final data set consisted of 447,666 observations for 16,379 individuals with running exercise records across 83 cities in China over a roughly 2-year time span. In our final data set, 35.99% (5896/16,379) of users were female and 64% (10,483/16,379) were male. We do not have access to the users’ age data; however, statistics from the focal fitness platform we analyzed suggest that their users’ age ranged predominantly from 18 to 50 years [14]. Furthermore, in terms of the users’ exercise behavior, the average exercise distance was around 6.61 km for the users in all the 83 cities, with an average exercise speed of around 8.74 km/h. At the weekly level, the average weekly exercise frequency was around 3.5 times, with an average distance of 7.13 km exercised each week.

We augmented the exercise behavior data with 2013 to 2014 air pollution information that includes city-hourly level measures of AQI and PM2.5 concentration (in µg/m^3^) from both the Chinese Ministry of Environmental Protection (CN-MEP; provided data for 83 cities) and the US Department of State (US-DOS; it provided data for 5 cities with embassies located in Beijing, Chengdu, Guangzhou, Shanghai, and Shenyang). Finally, for control purposes in the empirical analysis, we acquired the 2013 to 2014 National Oceanic and Atmospheric Administration Integrated Surface Database’s weather data for 83 cities that include city-hourly level measures of air temperature (ºC), dew point (ºC), wind speed (m/s), and wind direction (degrees). Table 1 shows the descriptive statistics for our data set at the individual user–level for both types of air quality indicator (ie, Air Quality Index-China [AQI-CN] and particulate matter 2.5-United States [PM2.5-US]) across the 5 cities of Beijing, Chengdu, Guangzhou, Shanghai and Shenyang, as well as across all 83 cities in our data set.

**Table 1:** Descriptive statistics (mean (SD)): individual-level data.

Variables			AQI-CN^a^ (5 cities)		AQI-CN (83 cities)		PM2.5-US^b^ (5 cities)
Exercise distance (km)			6.648 (4.423)		6.611 (4.413)		6.659 (4.438)
Air quality (PM2.5 or AQI)			89.983 (56.321)		88.339 (54.842)		63.515 (54.438)
50<air quality≤100			0.468 (0.499)		0.486 (0.500)		0.316 (0.465)
100<air quality≤150			0.177 (0.382)		0.182 (0.386)		0.105 (0.307)
150<air quality≤200			0.071 (0.257)		0.062 (0.242)		0.038 (0.190)
200<air quality≤300			0.046 (0.210)		0.037 (0.189)		0.021 (0.142)
300<air quality≤500			0.008 (0.089)		0.008 (0.092)		0.007 (0.081)
Air quality>500			—^c^		—		0.001 (0.023)
Exercise speed (km/h)			8.706 (5.714)		8.738 (12.398)		8.693 (4.067)
Temperature (ºC)			18.803 (9.181)		19.345 (9.013)		18.717 (9.226)
Dew point (ºC)			11.418 (11.470)		12.620 (10.678)		11.292 (11.485)
Wind speed (m/s)			27.417 (19.201)		26.815 (18.241)		27.530 (19.250)
**City**							
	Beijing	0.350 (0.477)		0.137 (0.343)		0.357 (0.479)	
	Chengdu	0.081 (0.273)		0.032 (0.175)		0.078 (0.269)	
	Guangzhou	0.175 (0.380)		0.068 (0.252)		0.167 (0.373)	
	Shanghai	0.370 (0.483)		0.144 (0.351)		0.375 (0.484)	
	Shenyang	0.024 (0.153)		0.009 (0.096)		0.023 (0.149)	
Observations			174,654		447,666		172,352
Number of individuals			7165		16,379		7146

^a^AQI-CN: Air Quality Index-China.

^b^PM2.5-US: particulate matter 2.5-United States.

^c^No recorded instance of air quality in this range.

### Air Pollution Information Sources

In China, mainstream air quality and pollution information source is the official pollutant standards index such as the AQI (ie, CN-MEP) communicated by government agencies. Beyond the mainstream information source, there are multiple alternative sources or channels from which individuals can acquire air pollution information. First, social media Weibo tweets can serve as an alternative form of information on air pollution [6]. There is a strong correlation between the Air Discussion Index constructed based on Weibo tweets and the actual measured PM in the air [6,15]. Second, for the 5 cities of Beijing, Chengdu, Guangzhou, Shanghai, and Shenyang, AQI and PM2.5 information provided by US-DOS can serve as an alternative information source corresponding to the mainstream one provided by CN-MEP. Third, we consider the case of mobile apps as an alternative information channel for air quality information [16]. Specifically, we focus on the China AQI mobile app (renamed as Air Matters in 2016), which was the pioneer for this category of apps and released their version 4.0 with over 160 cities coverage on May 10, 2013 [17]. This version added monitoring stations data from >800 monitoring stations in over 160 cities, constituting a substantial 33% increase in city level coverage of air quality information for mobile phone users. Fourth, there can be anchoring effects where individuals possess alternative or self-referent local information such as the prevailing levels of air pollution in a city [18], which may not be known to new visitors exercising in a location that is not their home city. Finally, individuals can access air pollution information from their connected peers on the focal fitness platform. Prior studies suggest that there can be social contagion in exercise behaviors [19] and transmission of information through these social connections [7].

### Statistical Analysis

#### Descriptive Analysis

**Figure 1:**
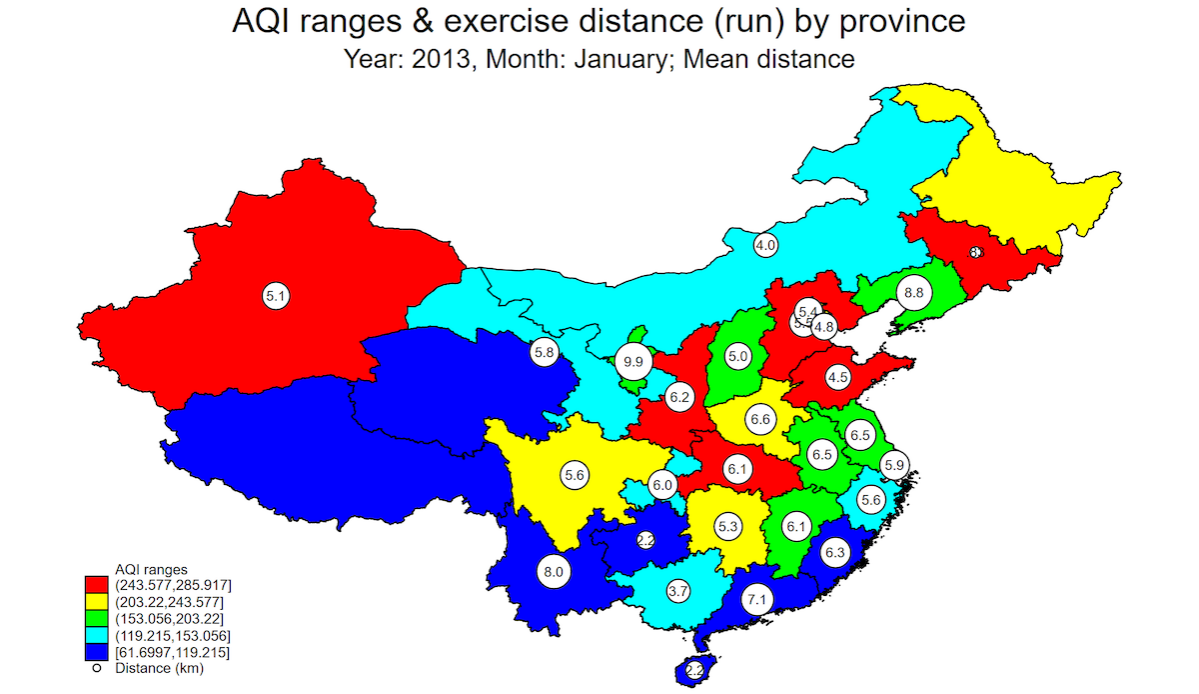
Mean of exercise distance. AQI: Air Quality Index.

**Figure 2:**
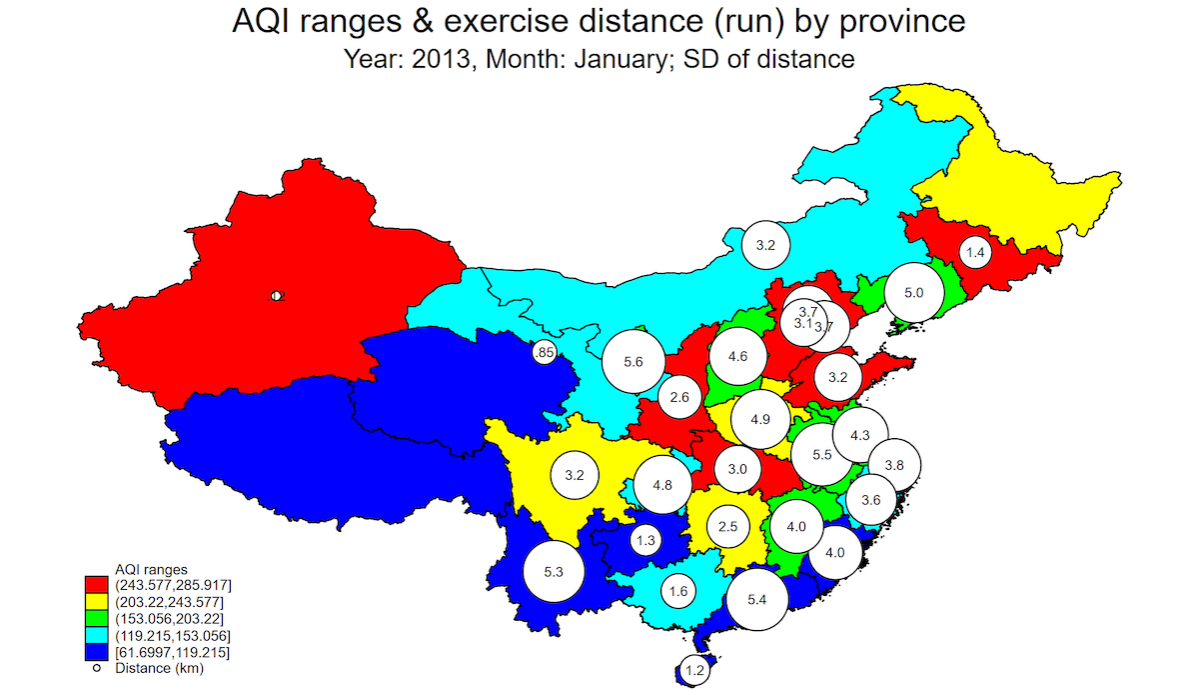
SD of exercise distance. AQI: Air Quality Index.

Figures 1 and 2 show a spatial plot of the means and SDs of exercise distances by 5 different bins or ranges of AQI-CN (ie, AQI data from CN-MEP) measures in January 2013 across China’s provincial-level administrative units. It is clear from Figure 1 that the mean running exercise distance is lower in the more heavily polluted provinces such as Hebei, Beijing, Tianjin, and Shandong (ie, the cluster of eastern provinces shaded in red), compared with the less polluted provinces along its southern coastline such as Guangdong, Fujian, Zhejiang, Shanghai, and Jiangsu. In terms of the SDs of exercise distance, Figure 2 shows that the variability of running exercise distance is higher in the less polluted provinces shaded in blue, light blue, and light green. Therefore, there is tentative descriptive evidence of individuals’ exercise-aversion behaviors in response to air pollution in various Chinese provinces.

#### Linear Fixed Effect Model

We first specified a linear panel model specification for our focal variable of interest, that is, running exercise distance at different units of analysis (ie, individual-hour, city-hour, or city-day levels). Subscript *i* in equation (1) below refers to either an individual or a city (in a specific province shown in Figure 1), and *t* refers to the time unit of either hour or day.

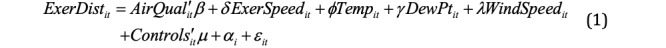



*ExerDist_it_* captures the running exercise distance for an individual or a city at either the hourly or aggregated daily level. *AirQual_it_* is a vector of air quality indicators measuring the extent of air pollution at either the hourly or daily level, specific to the city where an individual conducts the running physical activities or exercises. We allowed for nonlinear effects of air pollution in our model specification by discretizing pollution levels through the use of dummy variables, which is a commonly used approach in the literature [20]. Specifically, we included dummy variables that correspond to the different pollution thresholds based on the CN-MEP standard of AQI (AQI 0-50: excellent, 51-100: good, 101-150: lightly polluted, 151-200: moderately polluted, 201-300: heavily polluted, and >300: severely polluted). In addition, in our model, we accounted for an individual’s exercise speed (*ExerSpeed_it_*), city-hour or day level temperature (*Temp_it_*), dew point (*DewPt_it_*), and wind speed (*WindSpeed_it_*). Other controls include various dummy variables corresponding to the specific year, month, day of week, time of day, and city information during the incidence of an individual’s running exercise activity. Furthermore, we performed various placebo tests to verify that it is indeed the air pollution levels in each specific city and specific date and hour that are driving the specific aversion responses of individuals’ exercise behaviors (Table S1 in Multimedia Appendix 1).

#### Endogenous Treatment Effect Model

Daily and hourly variations (ie, the time unit of analysis for this study) of air pollutants can plausibly be considered as exogenous [21]. As air pollution is not randomly assigned to individuals, studies that attempt to compare aversion behaviors or health outcomes for populations exposed to varying pollution levels may not be adequately accounting for potential confounding factors. It is known that air quality is capitalized into real estate prices [22]. Thus, households with higher incomes or preferences for cleaner air are likely to sort into locations with better air quality. Failure to account for this sorting will lead to overestimates of the effects of pollution. Alternatively, pollution levels are typically higher in urban areas where there are often more educated individuals with better access to health care, leading to underestimates of the true effects of pollution.

To control for the potential endogeneity of mainstream air pollution indicators of PM2.5 and AQI measures, we used the endogenous treatment effect model [23] to model running exercise distance while accounting for the potential endogeneity (due to selection or sorting) of moderate to severe air pollution (ie, as indicated by the binary treatment dummy for AQI/PM2.5>150, ie, *AirPolluted*). The endogenous treatment effect model estimates an average treatment effect and the other parameters of a linear regression model for exercise distance, augmented with an endogenous binary-treatment variable for moderate to severe air pollution. The model specification is as follows:

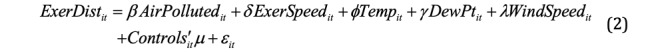

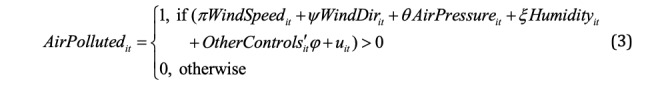





where *ε_it_* and *u_it_* are bivariate normal with mean zero and covariance matrix Σ.

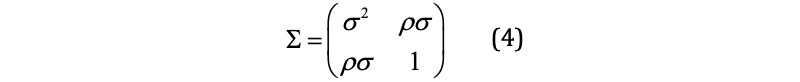



In our focal equation (2) of interest, *ExerDist_it_* captures the running exercise distance for an individual or a city at either the hourly or aggregated daily level. *AirPolluted_it_* is an individual or city-specific hourly or daily binary indicator for AQI-CN>150, that is, when air quality crosses into the moderate to severe air pollution range (ie, the same pollution threshold in literature [24], which have a substantial effect on individuals’ decision to purchase health insurance). We also account for an individual’s exercise speed (*ExerSpeed_it_*) and weather conditions such as temperature (*Temp_it_*), dew point (*DewPt_it_*), and wind speed (*WindSpeed_it_*). Appropriate control variables here include users’ point balance and exercise skill grade on the focal fitness platform, and fixed effect dummies corresponding to the specific calendar year, month, day of week, and time of day information. In the treatment equation (3), the *AirPolluted_it_* indicator is a probit function of city-hour or day level wind speed (*WindSpeed_it_*), wind direction (*WindDir_it_*), air pressure (*AirPressure_it_*), humidity (*Humidity_it_*), and other controls such as calendar month and day of week fixed effects. Equations (2) and (3) above are estimated by maximum likelihood estimation.

#### Regression Discontinuity

To further bolster the confidence of our estimate for the causal effect of air pollution on exercise aversion behaviors, we used the regression discontinuity method [25,26] as a robustness check to estimate the effect of air pollution severity crossing specific AQI categorical thresholds on individuals’ average running exercise distances at the city-hour level. We used the standard or continuity-based framework for regression discontinuity analysis, and specifically a sharp regression discontinuity design.

The regression discontinuity design can be used to isolate a treatment effect of interest from all other systematic differences between treated and control groups. Under appropriate assumptions [25,26], a comparison of individuals and cities for which the AQI indicators are barely below the moderate or severe pollution threshold and those for which the AQI indicators are barely above the same threshold will reveal the causal (local) effect of air pollution on exercise behaviors. If individuals and cities cannot systematically manipulate the air pollution indicators, observations just above and just below the cutoff will tend to be comparable in terms of all characteristics. Thus, right at the cutoff, the comparison is free of the complications introduced by systematic observed and unobserved differences between the treatment and control groups. Our regression discontinuity estimators are based on mean-squared error-optimal bandwidths. Further details of the regression discontinuity procedures and the related robustness checks are provided in Table S2 in Multimedia Appendix 2.

#### Moderation Effects Analysis: Alternative Information Sources

According to the Protection Motivation Theory, individuals’ motivation to engage in protective behaviors is influenced by 2 cognitive appraisal processes, that is, threat appraisal and coping appraisal [27]. In the context of our study, the Protection Motivation Theory is helpful to understand how individuals assess the risk of air pollution to their health and their perceived ability to cope with these risks through personal actions, such as adjusting their outdoor exercise activities. The availability of alternative information sources or channels, such as social media platforms, play an important role in shaping these appraisals [28]. The alternative information sources can provide additional narratives or evidence about air pollution, which can affect the cognitive process of threat and coping appraisals. Specifically, the alternative information sources may amplify or attenuate individuals’ perception of the threat (ie, the severity of the air pollution) and their belief in their capacity to take protective actions, such as modifying exercise routines. The interplay between alternative information sources and the cognitive appraisal processes emphasized by the Protection Motivation Theory underscores the necessity and significance of understanding the moderation effect of alternative information sources on the impact of mainstream air pollution information on individuals’ exercise aversion behaviors. We further conduct empirical analyses using an individual-level linear fixed effect model in equation (1) to evaluate if alternative sources of air pollution information have interaction effects with mainstream air pollution information, such that they either mitigate or accentuate (ie, substitute or complement) the impact of mainstream air pollution indicators on individuals’ exercise aversion behaviors.

First, we estimated if individuals’ exercise averting response to the mainstream AQI-CN pollution measures will be accentuated with increasing volumes of social media Weibo tweets of Chinese language phrases associated with air pollution. Social media can serve as an alternative form of salient confirmatory or reinforcement information on air pollution. Social media Weibo tweets can amplify official messages and contribute to a sense of urgency or provide practical coping strategies for individuals [6], which may heighten perceived severity and vulnerability and likely lead to a stronger motivation to engage in protective behaviors.

Second, we evaluated whether individuals located in the 5 cities of Beijing, Chengdu, Guangzhou, Shanghai, and Shenyang with the alternative PM2.5-US information respond more dramatically in exercise aversion behaviors, compared with those in the other 78 cities with no such alternative pollution information. Information source and credibility significantly influence how individuals interpret and act on information about air pollution [29], with the US-DOS being regarded as a reliable source, potentially strengthening individuals’ evaluation of the risk, and thus, promoting protective behaviors. We further estimated the effects using a neighboring matched-city approach (ie, matching Beijing to neighboring Tianjin, Chengdu to Chongqing, Guangzhou to Shenzhen, Shanghai to Hangzhou, and Shenyang to Dalian where there are no alternative PM2.5-US information).

Third, we evaluated if individuals’ exercise aversion responses to the mainstream AQI-CN pollution indicator are affected by the increased 160 cities coverage of air quality information in the China AQI mobile app. The AQI mobile app can provide real-time data on pollution levels and health-related alerts about the specific risks of current air quality levels, which can heighten individuals’ perception of the immediate risk and motivation to take protective action.

Fourth, we estimated whether individuals exercising in a location or city which is their home city alter their exercise behaviors to a larger extent during episodes of air pollution. Locals with anchoring knowledge for a specific home city have a coping advantage as they can refer to their experience to make informed decisions [30]. However, new visitors to a city may not possess local or home knowledge about the typical air pollution levels in a city and are likely to underestimate or overestimate the threat, affecting their coping appraisal and potential protective behaviors. This anchoring effect can therefore affect their perceived severity and coping appraisal.

Finally, we examined if individuals’ exercise aversion behaviors are moderated by the numbers and types of social connections on the focal fitness platform. Information from connected peers often carries important weight because it comes from a trusted and relatable source [31], so the air pollution information shared by connected peers may increase individuals’ awareness and perceived severity. We considered different types of social connections, that is, followers (incoming connections for the focal individual), followees (outgoing connections initiated by the focal individual), and mutual followers (individuals who are both the followers and followees for the focal individual).

### Ethical Considerations

All the data in this study were collected in a manner that ensures the anonymity and privacy of individuals in our sample. Each user in our sample has a unique anonymous user ID, which is randomly generated by the focal fitness platform and has no connection to the participant's actual identity. This study was reviewed and approved by the Departmental Ethics Review Committee of School of Computing at the National University of Singapore. The DERC Case No. is SOC-22-03.

## Results

Table 2 presents the model estimation results using the AQI-CN air quality indicators. Using a fixed effect model estimation approach at the individual-hour, city-hour, and city-day levels (ie, columns 1 to 3), we find significant evidence of individuals’ pollution aversion behavior in terms of decreasing their running exercise distances when air pollution worsened. Such aversion behaviors in terms of decreasing their running exercise distances were more sensitive or in larger magnitudes during incidences of moderate to severe air pollution. At the individual and city-hourly level, when the air was moderately polluted (ie, 150<AQI-CN≤200), running exercise distances decreased by about 0.24 km (*P*<.001) to 0.32 km (*P*<.001) relative to when air quality was excellent (ie, 0<AQI-CN≤50). When the air was severely polluted (ie, 300<AQI-CN≤500), running distances dropped by about 0.72 km (*P*=.03) at the city-hour level and 0.73 km (*P*=.002) at the city-day level. The results from the endogenous treatment effect models show general consistency with those from the linear fixed effect models (Table 2). At the individual and city-hourly level, running distances decreased by 0.48 km (*P*<.001) to 0.87 km (*P*=.07) in episodes of moderate to severe air pollution. Furthermore, the results of regression discontinuity estimations confirm that there was a decrease in running exercise distance of 0.29 km (*P*=.07, without covariates) at the AQI-CN cutoff value of 150 for moderately polluted air conditions (Table S3 in Multimedia Appendix 2). Generally, these results on the extent of exercise aversion in terms of decreased running distances also corroborate those from the linear fixed effect and endogenous treatment effect models.

For the moderation effects of alternative information sources, first, we find that as the volumes of tweets (in millions) about the Chinese-language words for “haze” and “face mask” (which reflect health concerns and self-protection measures [12,32]) increase, there was a larger extent of decrease in running exercise distances during episodes of moderate to heavy air pollution. In particular, a 1 SD increase in the volume of tweets about “haze” and “face mask” (ie, 0.21 and 0.026 million tweets respectively) was associated with a drop of about 0.09 km (*P*=.02) to 0.12 km (*P*<.001) in running distances during heavily polluted episodes (ie, 200<AQI-CN≤300; Table 3).

Second, we find that relative to those in the other 78 cities, individuals exercising in the 5 cities of Beijing, Chengdu, Guangzhou, Shanghai, and Shenyang with alternative PM2.5-US information did tend to decrease running distances to a larger extent by about 0.25 km (*P*<.001) during light to moderate air pollution episodes (Table 3). Similarly, using a neighboring matched-city approach (ie, matching Beijing to neighboring Tianjin, Chengdu to Chongqing, Guangzhou to Shenzhen, Shanghai to Hangzhou, and Shenyang to Dalian where there are no alternative PM2.5-US information), the results further confirm that individuals in the 5 cities with alternative PM2.5-US information reduced running distances by 0.19 km (*P*=.002) to 0.22 km (*P*<.001) even in instances of low to light air pollution (Table 3).

Third, considering the case of mobile apps as an alternative channel for air quality information, we find that individuals exercising after the increased 160 cities coverage of air quality information in the China AQI mobile app, did significantly decrease their running exercise distances by about 0.30 km (*P*=.02) and 0.60 km (*P*=.004) during heavy and severe air pollution episodes respectively (Table 3).

**Table 2:** Fixed effect and endogenous treatment models for Air Quality Index-China ranges (Chinese Ministry of Environmental Protection) [model coefficient (*P* value) reported].

Variables^a^	(1) FE^b,c^ (individual-hour level; 5 cities)	(2) FE^d^ (city-hour level; 5 cities)	(3) FE^e^ (city-day level; 5 cities)	(4) ET^c,f^ (individual level; 5 cities)	(5) ET^d^ (city-hour level; 5 cities)	(6) ET^e^ (city-day level; 5 cities)
AQI-CN^g,h^>150	—^i^	—	—	−0.479 (<.001)	−0.872 (.07)	−0.469 (.14)
50<AQI-CN≤100	−0.082 (.002)	−0.047 (.12)	−0.201 (.07)	—	—	—
100<AQI-CN≤150	−0.163 (<.001)	−0.174 (.01)	−0.254 (.08)	—	—	—
150<AQI-CN≤200	−0.242 (<.001)	−0.319 (<.001)	−0.291 (<.001)	—	—	—
200<AQI-CN≤300	−0.130 (.02)	−0.353 (.08)	−0.595 (.03)	—	—	—
300<AQI-CN≤500	−0.205 (.15)	−0.715 (.03)	−0.731 (.002)	—	—	—
Exercise speed (km/h)	0.029 (.08)	0.116 (.002)	0.092 (.10)	0.054 (.10)	0.117 (.005)	0.144 (.04)
Temperature (ºC)	−0.002 (<.001)	−0.002 (.05)	0.001 (<.001)	−0.002 (.04)	−0.001 (.32)	0.001 (.32)
Dew point (ºC)	0.001 (.01)	−0.000 (<.001)	−0.003 (.003)	−0.000 (<.001)	0.001 (<.001)	−0.001 (.32)
Wind speed (m/s)	−0.001 (.31)	−0.002 (.05)	−0.004 (<.001)	−0.002 (.04)	0.001 (.32)	−0.003 (.32)
Constant	6.551 (<.001)	6.889 (<.001)	6.656 (<.001)	4.215 (<.001)	7.079 (<.001)	6.257 (<.001)
Observations	174,654	33,617	3269	162,858	30,923	2978
*R*^2^ or log-likelihood	0.016	0.041	0.095	−519,371	−92,632	−6714
Number of individuals, n	7165	—^i^	—	7023	—	—

^a^Dependent variable: exercise distance (km).

^b^FE: fixed effect model.

^c^Controls for (1), (4): year, month, day of week, time of day, city dummies; individual’s reward points, and exercise skill grade.

^d^Controls for (2), (5): year, month, day of week, time of day dummies.

^e^Controls for (3), (6): year, month, day of week dummies.

^f^ET: endogenous treatment model.

^g^AQI-CN: Air Quality Index-China.

^h^Covariates for (AQI-CN>150) treatment indicator: wind speed, wind direction, air pressure, humidity, month and day of week dummies.

^i^Not applicable.

**Table 3:** Moderation effects of alternative information sources (part 1) [model coefficient (*P* value) reported].

Variables^a^	(1) Moderation factor: Weibo (*haze*; 雾霾); 83 cities (all including BJ^b^-CD^c^-GZ^d^-SH^e^-SY^f^)	(2) Moderation factor: Weibo *(face mask;* 口罩); 83 cities (all including BJ-CD-GZ-SH-SY)	(3) Moderation factor: particulate matter 2.5–United States; 83 cities (all including BJ-CD-GZ-SH-SY)	(4) Moderation factor: particulate matter 2.5–United States; 10 cities (BJ-CD-GZ-SH-SY, TJ^g^-CQ^h^-SZ^i^-HZ^j^-DL^k^)	(5) Moderation factor: 160 cities coverage in mobile app; 83 cities (all including BJ-CD-GZ-SH-SY)
50<air quality≤100	0.021 (.29)	0.036 (.10)	0.107 (<.001)	0.141 (<.001)	0.056 (.41)
100<air quality≤150	−0.044 (.09)	−0.032 (.25)	0.073 (.02)	0.032 (.56)	0.167 (.03)
150<air quality≤200	−0.118 (<.001)	−0.018 (.66)	−0.002 (.96)	−0.107 (.21)	−0.151 (.11)
200<air quality≤300	−0.018 (.70)	0.040 (.43)	−0.044 (.41)	−0.199 (.06)	0.207 (.05)
300<air quality≤500	−0.077 (.44)	−0.218 (.04)	−0.157 (.10)	−0.733 (.02)	−0.047 (.75)
50<air quality≤100 * moderation factor^l^	−0.002 (.97)	−0.712 (.21)	−0.194 (<.001)	−0.222 (<.001)	−0.019 (.81)
100<air quality≤150 * moderation factor^l^	0.049 (.59)	−0.346 (.61)	−0.248 (<.001)	−0.194 (.002)	−0.157 (.08)
150<air quality≤200 * moderation factor^l^	0.005 (.97)	−3.370 (<.001)	−0.250 (<.001)	−0.141 (.13)	0.025 (.82)
200<air quality≤300 * moderation factor^l^	−0.408 (.02)	−4.572 (<.001)	−0.095 (.22)	0.072 (.54)	−0.297 (.02)
300<air quality≤500 * moderation factor^l^	−0.411 (.12)	0.758 (.74)	−0.004 (.98)	0.546 (.10)	−0.597 (.004)
Moderation factor	−0.000 (<.001)	0.000 (<.001)	0.203 (.004)	0.194 (.009)	0.016 (.81)
Constant	6.216 (<.001)	6.197 (<.001)	6.065 (<.001)	5.918 (<.001)	5.269 (<.001)
Controls included^m^	Yes	Yes	Yes	Yes	Yes
Observations, n	444,193	444,193	447,666	258,648	88,341
*R* ^2^	0.015	0.015	0.015	0.016	0.025
Number of individuals, n	16,336	16,336	16,379	10,114	6989
Roy-Zellner *F* test	1.745	4.871	7.566	6.005	2.980
Prob>*F*	0.121	0.000187	4.16e-07	1.49e-05	0.0109

^a^Dependent variable: exercise distance (km).

^b^BJ: Beijing.

^c^CD: Chengdu.

^d^GZ: Guangzhou.

^e^SH: Shanghai.

^f^SY: Shenyang.

^g^TJ: Tianjin.

^h^CQ: Chongqing.

^i^SZ: Shenzhen.

^j^HZ: Hangzhou.

^k^DL: Dalian.

^l^Moderation factor in the interaction term. The specific moderation factor for each column is indicated in the second row for moderation factor.

^m^Controls: temperature, dew point, wind speed; year, month, day of week, time of day, city dummies; individual’s exercise speed, reward points, and exercise skill grade.

Fourth, in all 83 cities, individuals exercising in a city which is their home tend to be more responsive in decreasing the extent of running distances during polluted air. Such a moderation effect increased in magnitude across light to heavy and severe pollution episodes, with the drop in running distances ranging from 0.14 km (*P*=.003) to 0.40 km (*P*=.01) for individuals exercising in their home locations (Table 4). Similarly, individuals exercising in the 5 cities of Beijing, Chengdu, Guangzhou, Shanghai, and Shenyang exhibited this similar home-location moderation effect of 0.16 km (*P*=.01) to 0.60 km (*P*=.07) across light to heavy and severe pollution (Table 4).

Finally, we observe a negative moderation effect of social connections on the physical exercise aversion response to mainstream pollution information sources. Specifically, the numbers of followers, followees, and mutual connections for a focal individual accentuate the decrease in exercise behaviors during polluted air, even in good or lightly polluted conditions (Table 4). We note that the extent of this negative moderation effect of 1 additional social connection on individuals’ exercise behaviors during lightly polluted conditions (ie, a roughly 0.15 km drop; *P*=.001) is almost double that during good air conditions (ie, 0.08 km decrease; *P*=.02).

**Table 4:** Moderation effects of alternative information sources (part 2) [model coefficient (*P* value) reported].

Variables^a^	(1) Moderation factor: same exercise and home location; 83 cities (all including BJ^b^-CD^c^-GZ^d^-SH^e^-SY^f^)	(2) Moderation factor: same exercise and home location; 5 cities (BJ-CD-GZ-SH-SY)	(3) Moderation factor: social connections (followers); 5 cities (BJ-CD-GZ-SH-SY)	(4) Moderation factor: social connections (followees); 5 cities (BJ-CD-GZ-SH-SY)	(5) Moderation factor: social connections (mutual); 5 cities (BJ-CD-GZ-SH-SY)
50<air quality≤100	0.038 (.07)	−0.046 (.18)	−0.044 (.09)	−0.056 (.03)	−0.058 (.03)
100<air quality≤150	0.022 (.42)	−0.072 (.11)	−0.108 (.001)	−0.118 (<.001)	−0.120 (<.001)
150<air quality≤200	−0.085 (.02)	−0.282 (<.001)	−0.213 (<.001)	−0.225 (<.001)	−0.223 (<.001)
200<air quality≤300	0.001 (.98)	0.164 (.09)	−0.080 (.15)	−0.096 (.08)	−0.092 (.09)
300<air quality≤500	0.017 (.87)	0.266 (.37)	−0.142 (.31)	−0.136 (.33)	−0.151 (.28)
50<air quality≤100 * moderation factor^g^	−0.034 (.38)	−0.067 (.21)	−0.102 (.006)	−0.080 (.02)	−0.086 (.04)
100<air quality≤150 * moderation factor^g^	−0.142 (.003)	−0.161 (.01)	−0.154 (.002)	−0.149 (.001)	−0.160 (.004)
150<air quality≤200 * moderation factor^g^	−0.075 (.23)	0.042 (.63)	−0.077 (.11)	−0.052 (.24)	−0.068 (.17)
200<air quality≤300 * moderation factor^g^	−0.188 (.02)	−0.419 (<.001)	−0.136 (.06)	−0.107 (.09)	−0.138 (.08)
300<air quality≤500 * moderation factor^g^	−0.400 (.01)	−0.595 (.07)	−0.156 (.22)	−0.189 (.13)	−0.173 (.47)
Moderation factor	0.365 (.001)	0.592 (.049)	—^h^	—	—
Constant	5.874 (<.001)	6.284 (<.001)	6.547 (<.001)	6.551 (<.001)	6.550 (<.001)
Controls included^i^	Yes	Yes	Yes	Yes	Yes
Observations, n	447,666	174,654	174,654	174,654	174,654
*R* ^2^	0.015	0.016	0.016	0.016	0.016
Number of individuals, n	16,379	7165	7165	7165	7165
Roy-Zellner *F* test	2.271	4.723	2.162	2.693	2.013
Prob>*F*	0.0448	0.000261	0.0554	0.0195	0.0736

^a^Dependent variable: exercise distance (km).

^b^BJ: Beijing.

^c^CD: Chengdu.

^d^GZ: Guangzhou.

^e^SH: Shanghai.

^f^SY: Shenyang.

^g^Moderation factor in the interaction term. The specific moderation factor for each column is indicated in the second row for moderation factor.

^h^The main effect of social connections is included in the linear panel models, but its coefficient is dropped in the above fixed effect specification since the variable is nontime varying.

^i^Controls: temperature, dew point, wind speed; year, month, day of week, time of day, city dummies; individual’s exercise speed, reward points, and exercise skill grade.

## Discussion

### Principal Findings

We examine the extent to which individuals avoid exposure to polluted air during exercise activities, in response to different sources of air pollution information, and whether alternative information sources moderate the effect of mainstream air pollution information sources on such exercise behaviors. Our findings show that (1) individuals exhibited a decrease in running exercise behaviors by about 0.50 km in instances of moderate to severe air pollution and (2) the alternative sources of air pollution information, such as social media user–generated content about air pollution and social connections, complement the role of mainstream air pollution information in inducing individuals’ exercise-aversion behaviors.

Contrary to popular belief that people know how to cope with air pollution, such as reducing their outdoor physical exercising, our results show that individuals only reduce their running distances by about 0.50 km (or 7.5% on average across individuals in our sample) in instances of moderate to severe air pollution, and there is no causal evidence of reduced distances in instances of light air pollution. This averting response is less pronounced compared with that in previous studies based on different outcome metrics (eg, 8%-15% drop in outdoor facility attendances [21]; 14%-35% reduction in amount of cycling [33]). Individuals do seem to weigh the tradeoff of outdoor physical activities in the face of air pollution, but they did not exhibit a large extent of avoidance behaviors as expected. Drawing on the Protection Motivation Theory and related literature, there could be several potential explanations. First, individuals may not perceive air pollution as a severe health threat, or they have optimism bias about susceptibility to harm, which individuals consider themselves as less at risk than their peers regardless of their age and sex [34], and thus tend to view themselves as different from the susceptible group when assessing the risk of air pollution. If the perceived threat of air pollution is low, their motivation to change behavior such as reducing outdoor exercise will be low as well. Second, prior findings revealed that people who are healthy without asthma or cardiovascular disease are less likely to modify their behavior in the face of air pollution [35,36]. The participants in our sample, who are generally younger and in relatively good physical fitness [14,37], tend to underestimate the harmful impacts of air pollution during exercising, especially in the instances of light air pollution. Third, even if individuals recognize the risk of air pollution, they might feel incapable of changing their exercise routines due to personal constraints, such as lack of access to indoor exercise facilities or a strong preference for outdoor activities. Individuals might perform a cost-benefit analysis, either consciously or subconsciously, weighing the benefits of exercising outdoors against the potential health hazards of air pollution [38]. Individuals who perceive the benefits of outdoor exercise, such as enjoyment, social interactions, outweigh the potential negative impacts of exercising in polluted air, are less inclined to adjust their exercise behaviors.

Prior studies mainly focus on the air pollution information provided by official mainstream channels [3,4]. Our findings highlight the importance of alternative air pollution information sources beyond official channels in affecting individuals’ perception of air pollution risk and helping them make informed decisions on reducing air pollution exposure during physical activities. Furthermore, our findings reveal the differential moderation impacts of alternative information sources on individuals’ exercise behavioral responses during different air pollution conditions. In good to lightly polluted air conditions, the availability of alternative PM2.5-US information generally had the largest negative moderation impact. The PM2.5-US information offers an alternative perspective on air quality, potentially differing in methodology or perceived accuracy compared with local official sources based on AQI-CN. The existence of this alternative source allows individuals to cross-reference and assess the risks associated with air pollution more critically, which may amplify individuals’ perceived severity of and vulnerability to air pollution episodes. The enhanced perception can potentially increase their motivation to engage in protective behaviors. In moderately to heavily polluted conditions, social media Weibo tweets of “face mask” and “haze” had the most significant and largest negative-moderation impact on the effect of mainstream pollution information on exercise aversion. Social media posts can amplify the perceived severity of and personal vulnerability to air pollution because information and personal experiences shared within one’s social network can contribute to a heightened sense of risk. The visibility of collective attitudes and actions on social media strengthens the risk perception through social contagion [19], encouraging individuals to take protective behaviors. In the most severely polluted conditions, the availability of mobile app access to location-specific real-time air quality information had the most considerable negative moderation effect on exercise aversion behaviors. Given that many individuals use mobile phones to track their exercise activities, they are more likely to seek out air quality information using mobile apps before or during the exercises. Individuals use air quality mobile apps that provide real-time, localized air quality data, which can render the official air pollution information more personally relevant and actionable [39]. Such real-time and personalized data may lead to individuals to perceive the air pollution risk as more urgent and serious, prompting a stronger avoidance behavior in terms of reducing outdoor exercise.

Although prior literature has documented that exercise behaviors can be socially contagious [19], prior findings did not differentiate between the information transmission mechanism between different types of social connections. Our findings show that the efficacy of social connections as alternative sources of pollution information in affecting exercise aversion behaviors can rely on the type of social connection (ie, followers, followees, and mutual followers). Individuals tend to rely more on information about polluted air conditions from their followers and mutual followers and adjust the exercise aversion responses based on observational learning of peer behaviors [40]. Mutual followers, representing a stronger social bond, often share information perceived as more personalized or relevant. When individuals follow each other, the reciprocal ties imply a higher level of engagement and trust, which makes information shared by mutual followers seem more relevant and credible [31]. Individuals tend to view their followers as peers who are similar to themselves or part of their in-group. This perception can increase the trust in the information shared by these followers, as it may be more personalized, relevant to the user’s local context or specific interests, or validated by social proof from peers [41].

### Limitations and Future Work

While our study represents an initial endeavor to examine the extent to which individuals adjust their exposure to polluted air during physical exercise, and respond in real time to diverse information sources, we contend that this study has several limitations. First, the data set was collected from individuals using wearable devices or mobile fitness apps linked to a web and mobile fitness platform. We acknowledge that these individuals in our research sample are likely to have come from a selected sample who may be more predisposed to physical exercise activities compared with the general population. In this sense, the degree of air pollution aversion identified in our study is likely to be a conservative estimate. This is because enthusiasts of sports or fitness might not alter their physical activity routines, even during periods of air pollution. To mitigate this issue, researchers in future work could aim for a more representative sample of the general population or leverage field experiments. Second, regarding the phenomenon of web and mobile fitness platform and their gamification features, we acknowledge that individuals’ decisions in our data sample to engage in exercise activities could have been driven somewhat by the gamification elements on the platform. Nevertheless, in our empirical analysis at the individual user level, we control for these effects in our empirical models by including the user’s reward points and exercise skill grade as certified by the platform. Investigating the role of gamification elements on the platform in driving and sustaining behaviors aimed at health and fitness enhancement presents an intriguing research opportunity [42]. Third, future research could beneficially identify the threshold where the health benefits of physical activity in polluted conditions become outweighed by its adverse effects, taking into account various pollution levels and population demographics (eg, healthy individuals vs those with specific susceptibilities).

### Conclusions

In conclusion, this study is one of the firsts to uncover to what extent individuals weigh the exercise benefit-harm tradeoff to adapt their exercise activities in response to different air pollution information sources. The main strengths of this study include using comprehensive, disaggregate spatiotemporal data of both mainstream air pollution information sources and individuals’ detailed exercise behavioral records from wearable and mobile devices, examining individuals’ exercise-aversion behaviors in near real time on an hourly basis, and identifying and evaluating the impact of multiple alternative air pollution information sources. Furthermore, this study reveals the important role of different alternative sources of pollution information in inducing individuals’ exercise aversion responses.

Our findings offer insights for public health management and personal health decision-making from several aspects. First, policy makers may need to reassess the messaging and strategies used to communicate the risks associated with outdoor activities during episodes of high air pollution. Health authorities can use more personalized communication strategies, such as targeted messages for at-risk populations such as children, older adults, or those with respiratory conditions. To increase public awareness, reliance on traditional media and official air pollution alerts are not sufficient, public health authorities should leverage multitiered communication strategies that account for various demographics, including varying access to technology, as well as diversified information channels to keep the public engaged and informed about the dangers of high air pollution levels, especially in relation to outdoor activities. For instance, public health authorities may establish partnerships with tech companies and app developers to ensure the dissemination of air quality data is prompt, precise, and user-friendly. This can help create more tailored and engaging content that effectively promotes protective behaviors. Furthermore, the public awareness campaigns can be designed to capitalize on the virality and user engagement aspects of social media and mobile fitness platforms, utilizing these platforms to generate interactive and shareable content that facilitates the message dissemination. The dynamic nature of social media and mobile apps allow for real-time updates, which can keep the public informed during rapidly changing air quality situations, enhancing the public’s awareness and engagement in protective behaviors.

Second, our findings underscore the importance of disseminating location- and time-referent air quality information (eg, green space vs industrial area and 1-hour vs 24-hour AQI measurements) to better enable the public in evaluating the benefits and risks of health maintenance behaviors during air pollution episodes. Air quality can vary significantly within short distances and fluctuate throughout the day due to various factors such as traffic patterns, weather conditions, and industrial activities. By disseminating air quality information that is both location and time-referent, public health authorities can provide the public with a more comprehensive understanding of air pollution risks, which enables individuals to make better decisions about when and where to engage in outdoor activities. Specifically, by using mobile apps and related notifications, public health authorities can use location and time-specific air quality data and push real-time air quality updates to populations in high-risk areas. For instance, during a pollution spike in an industrial zone, authorities can advise residents on precautionary measures. Social media and mobile fitness platforms can serve as channels for sharing broader air quality trends and advisories. Such information can also be directly disseminated to individuals, citizens, and employees participating in increasingly popular wellness programs initiated and run by government agencies and commercial organizations (eg, insurance companies), and enabled by dedicated mobile apps or wearable devices for such programs.

Third, for the design of wearable devices and mobile apps, manufacturers of wearable devices and activity trackers can combine air pollution and health advisories with device features that monitor ambient air quality and users’ bio-physical indicators such as blood pressure that has been shown to be related to the extent of airborne pollutants. Mobile fitness app developers can also provide comprehensive real-time updates of mainstream and alternative information of air quality conditions through AQI measures, social connections, social media content, and geo-location advisories. For instance, with GPS information from mobile devices, app developers can issue time- and location-specific advisories based on real-time geo-locations of individuals and social connections engaged in physical activities or exercises, such as encouraging exercises in green spaces or parks, rather than alongside major roads with heavy vehicular traffic. With such real-time air quality data of different locations delivered through mobile apps or wearable devices, individuals can better assess the risks and make informed decisions about when and where to exercise outdoors. Future research should explore the long-term impact of repeated exposure to air pollution information and its impact on behavior over time, and explore whether individuals habituate to the alerts or if there are cumulative effects.

## Appendix

Multimedia Appendix 1 Robustness check using placebo tests.

Multimedia Appendix 2 Robustness check using regression discontinuity method.

## Abbreviations

AQI: Air Quality Index
AQI-CN: Air Quality Index-China
CN-MEP: Chinese Ministry of Environmental Protection
PM: particulate matter
