# Supplementary material for: The Impact of Air Pollution Information on Individuals’ Exercise Behavior: Empirical Study Using Wearable and Mobile Devices Data
Source: JMIR Mhealth Uhealth. 2024 Sep 10;12:e55207. doi: 10.2196/55207 (PMC11422738; doi:10.2196/55207)
Supplement: Multimedia Appendix 1 [file mhealth_v12i1e55207_app1.docx]

# Multimedia Appendix 1. Robustness check using placebo tests.

Using the linear fixed effect panel model specification in equation (1), we performed various placebo tests to verify that it is indeed the air pollution levels or information in each specific city and specific date and hour that are driving the specific aversion responses of individuals’ exercise behaviors.

First, we replaced the hourly AQI-CN measures of a focal city with those from a randomly selected different city for the same day. If the hourly AQI-CN readings embody some unobserved factors that are correlated with the outcome variable of exercise distance, using the AQI-CN readings from another random city to replace the true AQI-CN readings for the focal city in equation (1) would yield similar results to those from our main findings (Table 2 in main text), thus casting doubt on the exogeneity of the air pollution measures and the mechanism driving the aversion behaviors.

Second, we replaced the hourly AQI-CN measures of a focal city for a specific day with those in the same city but from randomly selected different days. Similar to the prior case, if using the hourly AQI-CN readings from a random different day to replace the true AQI-CN readings for the focal city yields significant model estimation results similar to those in Table 2 in main text, this will again raise concerns on the true mechanisms or unobserved omitted factors driving the exercise aversion behaviors.

Reassuringly, results of the above two placebo tests shown in Table S1 reveal that none of the estimated coefficients for the AQI-CN pollution threshold indicators are significant at the 5% level across three different units of analyses at the individual, city-hour and city-day levels. This thus largely dismisses the concern on the potential endogeneity of air pollution due to omitted variables in our empirical analysis. Importantly, this affirms the effect of air pollution severity, based on mainstream information sources in each specific city, influencing individuals’ exercise aversion behaviors.

Table S1. Placebo tests for Air Quality Index-China ranges (Chinese Ministry of Environmental Protection)

| Placebo | Random different city, Same date | | | Same city, Random different date | | |
| --- | --- | --- | --- | --- | --- | --- |
| Model | (1): FE^b,c^ | (2): FE^b,d^ | (3): FE^b,e^ | (4): FE^b,c^ | (5): FE^b,d^ | (6): FE^b,e^ |
| Level | Individual | City-hour | City-day | Individual | City-hour | City-day |
| Variables^a^ | 5 cities | 5 cities | 5 cities | 5 cities | 5 cities | 5 cities |
|  |  |  |  |  |  |  |
| 50 < AQI-CN <= 100 | 0.053 | 0.086 | 0.090 | −0.009 | −0.024 | 0.089 |
|  | (.021)^f^ | (.47) | (.61) | (.71) | (.39) | (.35) |
| 100 < AQI-CN <= 150 | 0.028 | 0.039 | −0.063 | 0.011 | −0.041 | 0.006 |
|  | (.59) | (.87) | (.85) | (.73) | (.41) | (.96) |
| 150 < AQI-CN <= 200 | 0.020 | 0.041 | 0.363 | −0.033 | −0.021 | 0.072 |
|  | (.76) | (.90) | (.35) | (.40) | (.63) | (.61) |
| 200 < AQI-CN <= 300 | 0.083 | 0.022 | 0.178 | 0.062 | 0.021 | 0.267 |
|  | (.27) | (.94) | (.69) | (.15) | (.71) | (.37) |
| 300 < AQI-CN <= 500 | −0.157 | 0.239 | 0.062 | 0.126 | 0.187 | 0.222 |
|  | (.34) | (.28) | (.92) | (.09) | (.17) | (.30) |
| Exercise speed (km/h) | 0.094 | 0.188 | 0.352 | 0.028 | 0.115 | 0.076 |
|  | (<.001) | (<.001) | (<.001) | (.09) | (.005) | (.39) |
| Temperature (C) | −0.002 | −0.002 | 0.003 | −0.001 | −0.002 | 0.000 |
|  | (.04) | (.04) | (.13) | (<.001) | (.04) | (<.001) |
| Dew point (C) | 0.002 | 0.001 | −0.001 | 0.001 | 0.001 | −0.001 |
|  | (.04) | (.32) | (.32) | (.001) | (<.001) | (<.001) |
| Wind speed (m/s) | 0.000 | −0.000 | 0.001 | −0.000 | −0.001 | −0.004 |
|  | (<.001) | (<.001) | (.80) | (<.001) | (.32) | (.42) |
| Constant | 7.038 | 6.122 | 3.310 | 7.133 | 6.788 | 6.503 |
|  | (<.001) | (<.001) | (.002) | (<.001) | (<.001) | (<.001) |
|  |  |  |  |  |  |  |
| Observations | 75,903 | 15,045 | 2,319 | 170,777 | 32,990 | 3,256 |
| *R*^2^ | 0.019 | 0.049 | 0.116 | 0.010 | 0.032 | 0.057 |
| Number of individuals, n | 3,157 | — | — | 7,145 | — | — |

1. Dependent variable: exercise distance (km).
2. FE: Fixed effect model.
3. Controls for (1), (4): year, month, day of week, time of day, city dummies; individual’s reward points and exercise skill grade.
4. Controls for (2), (5): year, month, day of week, time of day dummies.
5. Controls for (3), (6): year, month, day of week dummies.
6. *P* values are reported in parentheses.
