# Supplementary material for: The Impact of Air Pollution Information on Individuals’ Exercise Behavior: Empirical Study Using Wearable and Mobile Devices Data
Source: JMIR Mhealth Uhealth. 2024 Sep 10;12:e55207. doi: 10.2196/55207 (PMC11422738; doi:10.2196/55207)
Supplement: Multimedia Appendix 2 [file mhealth_v12i1e55207_app2.docx]

# Multimedia Appendix 2. Robustness check using regression discontinuity method.

The regression discontinuity design can be used to isolate a treatment effect of interest from all other systematic differences between treated and control groups. Under appropriate assumptions [1, 2], a comparison of individuals and cities for which the PM2.5 or AQI indicators are barely below the moderate or severe pollution threshold and those for which the PM2.5 or AQI indicators are barely above the same threshold will reveal the causal (local) effect of air pollution on exercise behaviors. If individuals and cities cannot systematically manipulate the air pollution indicators, observations just above and just below the cutoff will tend to be comparable in terms of all characteristics. Thus, right at the cutoff, the comparison is free of the complications introduced by systematic observed and unobserved differences between the treatment and control groups.

Figure S1 illustrates the scatter plots and regression discontinuity plots for the outcome variable of running exercise distance plotted against the explanatory variable of AQI-CN measures delineated by the various pollution severity thresholds. For example, in the case of AQI-CN, vertical lines in the sub-panels of Figure S1 indicate threshold cutoffs at 50 (for good air quality), 100 (for lightly polluted), 150 (for moderately polluted), 200 (for heavily polluted) and 300 (for severely polluted). While most regression continuity plots show some visual evidence of discontinuities in the running exercise distances when AQI-CN crossed various threshold cutoffs with increasingly severe air pollution, only the threshold cut-off value of 150 show a consistent distinct drop in running distances when going from below to above the 150 cut-offs in Figure S1.

Consistent with the moderate air pollution threshold cutoff of 150 used in the endogenous treatment effect model, we therefore consider the treatment is effected if (AQI-CN>150). Using a fourth-order polynomial regression line and quantile-spaced bins (i.e., non-overlapping intervals that partition the entire support of the air pollution indicator score, all containing (roughly) the same number of observations within each treatment assignment status), we graphically represent the regression discontinuity analysis by plotting a subset and all of the observations used for estimation and inference. The numbers of bins are chosen so that the overall variability of the binned means mimics the overall variability in the raw scatter plot of the data. Figure S2 shows the local and global polynomial fit (represented by a line) of running exercise distance by different AQI-CN pollution indicators and the local sample means within each bin (represented by dots). The polynomial regression lines in Figure S2 shows a distinct kink and drop in running exercise distances when going from below to above the 150 AQI-CN cut-offs, thus providing graphical evidence of the local causal effect of moderate air pollution on decreased exercise behaviors.

To conduct formal estimation and inference with the regression discontinuity analysis, we first used local polynomial methods with order one, a triangular kernel function and an optimal bandwidth in a mean-squared error sense to estimate the desired polynomials using only the observations near the AQI-CN cut-offs of 150. We then used the same bandwidth for inference purposes but with robust bias-corrected standard errors of the estimated focal coefficient to account for the effects of potential misspecification due to a large bandwidth as well as the additional sampling error introduced by such a correction. This robust bias correction has the advantage that the same observations in our data set can be used for both estimation and inference, thus leading to more powerful statistical tests and methods. Importantly, we also cluster the standard errors of the regression discontinuity estimator by city ids to account for potential correlation in exercise behaviors in the same city.

Besides the local polynomial fit that includes only the running variable (ie, AQI-CN measure) as a regressor, we also conducted additional analyses by including a set of predetermined covariates in the local polynomial regressions. These additional covariates for the regression discontinuity estimator include exercise speed, temperature, dew point, wind speed, calendar year and month, day of week and time of day dummies. In general, for our focal treatment cut-off of 150 for AQI-CN, the results of the estimated effect of moderate air pollution on running exercise distance remain largely consistent with and without adding covariates to the local polynomial regressions. This is reassuring in the sense that the included covariates are likely truly predetermined and the unadjusted estimator as well as the covariate-adjusted estimator are estimating the same parameter.

To assess the robustness of our results from the regression discontinuity approach, we evaluated the plausibility of its identification assumptions. First, we checked the continuity of the score density (ie, for AQI-CN) around the focal cut-off of 150 to assess if the number of observations below this cut-off is significantly different from that above the cut-off. In the absence of sorting and manipulation by individuals or cities on the air quality indicator scores that they receive, the number of treated observations just above the cut-off should be similar to that of control observations below it. Figure S3 shows that there is no statistical evidence of manipulation at the cut-off of 150 for AQI-CN, and thus offer support in favor of the regression discontinuity design.

Second, we conducted a series of falsification test that involves examining whether, near the cut-off of 150, treated observations are similar to control ones in terms of observable characteristics. If individuals or cities lack the ability to precisely manipulate the air quality indicator score value they receive, other than their treatment status, individuals or cities just above and just below the cut-off of 150 should be similar in all observable characteristics that could not have been affected by the treatment. Indeed, results of regression discontinuity analyses in Table S2 for the predetermined covariates (ie, individuals’ total exercise distance, points balance, exercise skill grade, exercise speed, as well as cities’ temperature, dew point, wind speed, wind direction and air pressure) affirm that there are no significant discontinuities in any covariate values around the 150 cut-off for AQI-CN.

Third, we performed another falsification check based on *placebo* alternative cut-off values beside 150. Specifically, we replaced the focal cut-off value of 150 for AQI-CN (corresponding to the cut-off for moderate air pollution) by another value at which the treatment status does not really change (eg, 100 corresponding to light air pollution) to then perform estimation and inference using this alternative cut-off point. Results in Table S3 show that for other *placebo* alternative treatment cut-offs (eg, 50, 100, 200, 300), there is no significant evidence of the causal effect of air pollution indicators on exercise aversion behaviors at the city-hour level. This is also the case when we repeated the regression discontinuity falsification analyses at the individual and city-day levels.

**References**

1. Imbens GW, Lemieux T. Regression Discontinuity Designs: A Guide to Practice. J. Econometrics 2008; 142(2): 615-635. doi: 10.1016/j.jeconom.2007.05.001
2. Lee DS, Lemieux T. Regression Discontinuity Designs in Economics. J. Econ. Lit. 2010; 48(2): 281-355. doi: 10.1257/jel.48.2.281

Figure S1. Scatter plot and regression discontinuity plots of city-hour level exercise distance by Air Quality Index-China ranges (for 5 cities: Beijing, Chengdu, Guangzhou, Shanghai, Shenyang).

|  |  |
| --- | --- |
|  |  |
|  |  |

Figure S2. Regression discontinuity plot at Air Quality Index-China=150 treatment cut-off.

| (A) Segmented AQI Range   | (B) Entire AQI Range   |
| --- | --- |

Figure S3. Manipulation or Density test for Air Quality Index-China running variable near treatment cut-off.

| (A) Segmented AQI Range    t = -1.002, *p* > \|t\| = 0.316 | (B) Entire AQI Range    t = -1.002, *p* > \|t\| = 0.316 |
| --- | --- |

Table S2. Covariate balance tests on pre-determined covariates near Air Quality Index-China=150 treatment cut-off (Local polynomial and partitioning regression methods).

|  |  |  | Robust Inference | |  |
| --- | --- | --- | --- | --- | --- |
| Variable | MSE-Optimal Bandwidth | RD Estimator | *p*-Value | Confidence  Interval | Number of Observations |
| Total distance (km) | 38.814 | −64.681 | 0.265 | [−178.48, 49.12] | 33617 |
| Points balance | 73.983 | −1445.000 | 0.336 | [−4390.29, 1500.32] | 33617 |
| Exercise skill grade | 47.893 | −0.180 | 0.281 | [−0.51, 0.15] | 33617 |
| Exercise speed (km/h) | 49.661 | 0.117 | 0.658 | [−0.40, 0.64] | 33617 |
| Temperature (C) | 20.124 | −6.042 | 0.458 | [−22.01, 9.92] | 33617 |
| Dew point (C) | 29.099 | −3.977 | 0.765 | [−30.04, 22.09] | 33617 |
| Wind speed (m/s) | 49.628 | 0.229 | 0.966 | [−10.31, 10.77] | 33617 |
| Wind direction (degs) | 39.448 | −5.504 | 0.709 | [−34.43, 23.42] | 33611 |
| Air pressure (hPa) | 14.381 | 19.143 | 0.197 | [−9.96, 48.24] | 16567 |

Table S3. Regression discontinuity results and placebo tests on exercise distance at alternative Air Quality Index-China treatment cut-offs (Local polynomial and partitioning regression methods).

|  |  |  | Robust Inference | |  |  |  |
| --- | --- | --- | --- | --- | --- | --- | --- |
| Alternative  Cut-off | MSE-Optimal Bandwidth | RD Estimator | *p*-Value | Confidence  Interval | Obs.  Left | Obs.  Right | With Covariates^a^ |
| 50 | 16.220 | 0.193 | 0.324 | [−0.190, 0.576] | 7631 | 25986 | No |
| 100 | 28.526 | −0.055 | 0.819 | [−0.531, 0.420] | 22665 | 10952 | No |
| 150 | 58.281 | −0.288 | 0.066 | [−0.595, 0.019] | 28720 | 4897 | No |
| 200 | 69.831 | −0.008 | 0.960 | [−0.322, 0.306] | 31138 | 2479 | No |
| 300 | 56.974 | −0.335 | 0.776 | [−2.639, 1.969] | 33125 | 492 | No |
| 50 | 12.843 | 0.180 | 0.419 | [−0.256, 0.615] | 7631 | 25986 | Yes |
| 100 | 24.007 | 0.003 | 0.990 | [−0.377, 0.382] | 22665 | 10952 | Yes |
| 150 | 69.258 | −0.201 | 0.152 | [−0.476, 0.074] | 28720 | 4897 | Yes |
| 200 | 78.335 | 0.090 | 0.439 | [−0.138, 0.318] | 31138 | 2479 | Yes |
| 300 | —^b^ | — | — | — | — | — | Yes |

1. Covariates include exercise speed, temperature, dew point, wind speed, calendar year and month, day of week and time of day dummies.
2. RD model with covariates and at alternative cut-off of AQI=300 cannot be estimated.
